# Integrative multi-omics Mendelian randomization reveals key lipid metabolism genes as therapeutic targets for diabetic nephropathy pathogenesis

**DOI:** 10.1080/0886022X.2026.2671460

**Published:** 2026-05-31

**Authors:** Shan Liu, Yan Xia, Lin Ding, Mingxia Zhang, Wangzhen Wang, Junying Zhou, Lunzhi Liu

**Affiliations:** Department of Nephrology, Hubei Provincial Clinical Medical Research Center for Nephropathy, Minda Hospital of Hubei Minzu University, Hubei Minzu University, Enshi, Hubei, China

**Keywords:** Diabetic nephropathy, lipid metabolism, Mendelian randomization, ceramide synthase 2, mediator complex subunit 27, group IB phospholipases A2

## Abstract

Dysregulated lipid metabolism contributes to diabetic nephropathy (DN), but the genetic links remain unclear. This study identified lipid metabolism genes associated with DN risk using summary-data-based Mendelian randomization (SMR). SMR screened 757 lipid metabolism genes by integrating large-scale blood methylation quantitative trait locus (mQTL; *n* = 1,980), expression QTL (eQTL; *n* = 31,684), and protein QTL (pQTL; *n* = 54,219) datasets with FinnGen genome-wide association study (5,042 cases/79,344 controls), followed by validation in GCST005881 (5,908 cases/4,967 controls). Functional enrichment, protein-protein interaction, and mQTL-eQTL integration were performed. Nephroseq data were used to examine tissue-level expression-renal function correlations. External bulk and single-cell transcriptomic datasets were analyzed to assess stage- and cell-type-specific expression. *CERS2, MED27,* and *PLA2G1B* were prioritized as DN-associated risk genes. Genetically predicted higher expression of *CERS2* (OR = 1.246, p_SMR = 0.027) and *MED27* (OR = 1.551, p_SMR = 0.032) increased DN risk. Methylation at cg26058502 (*CERS2:* OR = 0.604, FDR = 1.61 × 10–23) and cg13628444 (*MED27*: OR = 0.662, FDR = 3.82 × 10^−10^) demonstrated negative regulatory effects on gene expression. *PLA2G1B* was linked to increased risk at methylation (cg16396488: OR = 1.369, p_SMR = 7.82 × 10^−4^) and protein (OR = 3.057, p_SMR = 0.005) levels. However, in the Nephroseq database, higher expression of all three genes correlated with better kidney function. External bulk transcriptomics showed transient upregulation of *CERS2* and *MED27* in early DN but not advanced stages. *PLA2G1B* exhibited no differential expression or association with renal function. Single-cell analysis demonstrated broad detection of *CERS2* and *MED27* across renal cell types with subset enrichment, whereas *PLA2G1B* expression was sparse, with no evident disease-associated shifts. These findings indicate discordance between genetically inferred risk effects and observed expression patterns, suggesting complex regulatory mechanisms.

## Introduction

Diabetic nephropathy (DN), the leading cause of end-stage renal disease worldwide, is characterized by progressive proteinuria, glomerular hypertrophy, and renal function decline [1–3]. While current therapies targeting glycemic control and RAAS inhibition have shown efficacy, emerging treatments like SGLT2 inhibitors and GLP-1 agonists demonstrate variable patient responses. This pattern highlights the disease’s complex pathogenesis, involving oxidative stress, metabolic dysregulation, and ferroptosis [4–6], and underscores the clinical heterogeneity complicating the development of personalized therapies and creating an urgent need for novel mechanistic insights.

Lipid metabolic disturbances, particularly in triglyceride, cholesterol, and sphingolipid pathways, have emerged as central players in DN progression, implicated in processes such as detrimental lipid reprogramming and ferroptosis[7–9]. Paradoxically, while statins improve cardiovascular outcomes, they may exacerbate renal lipid deposition and fibrosis in some contexts [10]. Encouragingly, recent discoveries point to promising therapeutic targets, including SIRT6, which modulates glycolipid metabolism [11], and mechanisms by which SGLT2 inhibitors reduce renal lipid accumulation [12]. These findings position lipid metabolism as a critical, yet incompletely understood, axis in DN pathogenesis.

Despite this recognition, the specific contributions of numerous lipid metabolism-related genes in DN, and how their activity might be epigenetically regulated (e.g. by DNA methylation) to influence disease risk, remain largely uncharacterized. Identifying which of these genes hold potential as genetically validated therapeutic targets is therefore a key challenge that traditional drug discovery methods struggle to address efficiently due to DN’s complexity [13]. Integrative multi-omics approaches, particularly Mendelian randomization (MR) combining GWAS data with molecular QTLs (mQTLs, eQTLs, pQTLs), offer a powerful strategy for strengthening biological inference and prioritizing candidate genes [14–16].

Therefore, this study employed Summary-data-based Mendelian Randomization (SMR) to investigate the potential impact of 757 pre-selected lipid metabolism-related genes on DN risk. This approach is strengthened by integrating methylation, expression, and protein QTLs, validating key signals in large cohorts, linking methylation to expression through cross-omics SMR, and confirming tissue relevance using Nephroseq transcriptomic data. This integrative framework aims to uncover genetically supported molecular mechanisms underlying DN and provide potential therapeutic targets.

## Methods

### Study design

In this study, SMR analyses were conducted to identify lipid metabolism–related genes potentially linked to DN. The FinnGen and GCST005881 cohorts were used for discovery and replication, respectively. Blood-derived mQTL, eQTL, and pQTL data were integrated with DN GWAS results, and associations were evaluated using SMR and heterogeneity-in-dependent-instruments (HEIDI) tests. Significant loci (p_SMR < 0.05, p_SMR_multi < 0.05, p_HEIDI > 0.01) underwent colocalization and mQTL-eQTL SMR analyses. Tissue-level validation was performed using the Nephroseq database to examine associations between gene expression and renal function indicators. External bulk and single-cell transcriptomic datasets were analyzed to evaluate gene expression patterns in different disease stages and cell types. The workflow is presented in Figure S1.

### Data resources

Using the keyword ‘Lipid Metabolism’ in the Reactome database (https://reactome.org/PathwayBrowser/#/R-HSA-556833&DTAB=MT), 757 genes related to lipid metabolism were retrieved.

The GWAS data for the discovery dataset on DN were sourced from the FinnGen consortium, encompassing 5,042 cases and 79,344 controls. Replication was conducted using the GCST005881 cohort, comprising 5,908 cases and 4,967 controls.

Summary data on blood mQTLs originated from a meta-analysis involving two European cohorts: the Brisbane Systems Genetics Study (*n* = 614) and the Lothian Birth Cohorts (*n* = 1,366) [17]. Data on blood eQTLs came from the eQTLGen Consortium, which comprised genetic information on blood gene expression from 31,684 individuals [18]. Summary data on blood pQTLs were sourced from research conducted by Sun et al. which included 54,219 participants [19]. Dataset characteristics are summarized in Supplementary Table S1. All summary statistics used for SMR analyses were extracted from previously published studies that had already undergone appropriate ethical approval processes.

### SMR analysis

The SMR approach was utilized to assess the association between lipid metabolism gene methylation, expression, and protein abundance with DN. Gene expression, methylation, and protein levels derived from eQTL, mQTL, and pQTL data were treated as continuous traits. SMR tested the association between genetically predicted levels (instrumented by cis-QTLs) and DN risk using summary-level data. Effect estimates (β_SMR) were derived using a Wald-type ratio (β_SNP–GWAS/β_SNP–QTL). Odds ratios (ORs) were calculated as exp(β_SMR), representing the change in DN risk per unit increase in the genetically predicted molecular trait. OR > 1 indicates increased risk, whereas OR < 1 indicates decreased risk. Compared to traditional MR analysis, SMR employs advanced analytical methods to combine eQTL data with complex trait associations. This approach serves as an effective strategy for identifying priority candidate genes and can attain greater statistical power, particularly when the exposure and outcome data are from multiple independent large samples. In this study, the top associated cis-QTL were selected within a genomic window of ±1000 kb centered around the corresponding gene, using a p-value threshold of 5.0 × 10^−8^. The SNPs with allele frequency differences exceeding a predefined threshold (set at 0.2 in this study) between any pairwise data sets, including the linkage disequilibrium reference sample, QTL summary data, and outcome summary data, were excluded. Effect alleles were aligned between QTL and GWAS datasets using the SMR software. The maximum proportion of SNPs with allele frequency differences allowed is set to the default value of 0.05.

The study applied a multi-SNP SMR method that considers multiple SNPs at QTL loci [20]. The HEIDI test used a p_HEIDI threshold of >0.01 to distinguish pleiotropy from linkage. False discovery rate (FDR)-adjusted p-values were calculated for SMR results. Given the combined use of SMR significance and HEIDI filtering, and to avoid overly conservative filtering, results meeting the criteria of p_SMR_multi < 0.05, p_SMR < 0.05, and p_HEIDI > 0.01 were retained as valid SMR associations and subsequently subjected to colocalization analysis [21]. To further investigate the associations between mQTLs and eQTLs, mQTLs were treated as the exposure and eQTLs as the outcome in SMR analysis. FDR correction was applied to control for multiple comparisons in the mQTL-eQTL analysis. All SMR and HEIDI analyses were performed using the SMR software (version 1.3.1) [17].

To establish instrument validity and define valid SMR associations, genes were first filtered based on significant SMR associations (p_SMR < 0.05 and p_SMR_multi < 0.05) and absence of heterogeneity (p_HEIDI > 0.01). Following this instrument validity filtering, subsequent gene prioritization emphasized cross-omics support and significant mQTL-eQTL associations. This prioritization strategy was independent of formal posterior probability-based colocalization inference, as Bayesian colocalization can be overly conservative and prone to false negatives in regions with complex linkage disequilibrium [22]. Therefore, consistent multi-layer molecular evidence was utilized as an orthogonal biological filter to ensure the robustness of our candidates.

### Colocalization analysis

To strengthen the interpretation of associations between lipid metabolism and DN, colocalization analysis was conducted using the coloc R package to determine if the observed signals shared common genetic variants across multiple omics layers. The analysis centered on the colocalization region windows within a range of ±1000 kb [23]. Colocalization results were interpreted based on the posterior probability (PP) for five hypotheses: no association with either trait (H0), association only with gene expression (H1), association only with disease risk (H2), associations with both traits but driven by different variants (H3), and associations with both traits driven by the same variant (H4). PP.H4 > 0.5 at p12 = 5 × 10^−5^ indicated colocalization between QTL and GWAS signals, suggesting that a shared genetic variant influences both the QTLs and the disease trait.

### Enrichment analysis

Gene Ontology (GO) and Kyoto Encyclopedia of Genes and Genomes (KEGG) enrichment analyses were performed using the R package clusterProfiler. The combined gene set obtained from eQTL and pQTL analyses served as input. GO enrichment included three categories: biological process, molecular function, and cellular component. KEGG pathway enrichment analysis excluded pathways classified as human diseases. All enrichment p-values were adjusted using the Benjamini–Hochberg method to control for false discovery rate (FDR), and pathways with adjusted *p* < 0.05 were considered significant.

### Protein–protein interaction (PPI) network analysis

The STRING database (https://string-db.org/) was employed to analyze potential protein–protein interactions among the SMR-identified genes and to construct the PPI network. The resulting network was imported into Cytoscape (version 3.9.1) for visualization and further analysis. Additionally, to expand the interaction network and predict shared biological functions, GeneMANIA (http://www.genemania.org) identified genes functionally related to the target set based on co-expression, co-localization, and physical interaction data.

### Nephroseq database analysis

To examine the associations between candidate genes and renal function, correlation analyses were conducted using the Nephroseq v5 database (https://www.nephroseq.org), which contains transcriptomic profiles derived from human kidney tissue samples of DN patients [24]. Gene expression matrices and corresponding clinical parameters, including serum creatinine, glomerular filtration rate (GFR), and proteinuria, were extracted for analysis. Data were preprocessed according to Nephroseq normalization protocols, and missing or duplicate probe values were removed. Statistical analyses were performed to assess correlations between gene expression and renal function indicators using Pearson’s correlation within the Nephroseq platform.

### External transcriptomic and single-cell validation

To evaluate the expression patterns and potential stage- and cell-type-dependent behavior of prioritized genes, external bulk and single-cell transcriptomic datasets were analyzed. Bulk transcriptomic datasets GSE142025 and GSE30529, both derived from human DN kidney tissue, were obtained from the Gene Expression Omnibus (GEO). In GSE142025, samples were classified into control (*n* = 9), early DN (*n* = 6), and advanced DN (*n* = 22) groups after preprocessing and curation of clinical annotations [25]. Gene expression distributions across groups were visualized using violin plots. In GSE30529, associations between gene expression and renal function were evaluated using GFR as a clinical indicator based on integrated expression and clinical metadata [26].

Single-cell RNA sequencing (scRNA-seq) data were obtained from GSE131882, including kidney cortex samples from three controls and three patients with early DN [27,28]. Data were processed in R using Seurat. Genes expressed in fewer than three cells and cells with fewer than 200 detected genes were excluded. ENSEMBL IDs were converted to gene symbols, and duplicated or unmatched entries were removed. All samples were then merged for downstream analysis. Cells with 200–5000 detected genes and mitochondrial gene proportion <20% were retained. Data were normalized using the LogNormalize method, and highly variable genes were identified using the vst approach. During scaling, sequencing depth and mitochondrial content were regressed out. Principal component analysis was used for initial dimensionality reduction, followed by Harmony correction to account for sample-related variation. Cells were clustered using the Louvain algorithm and visualized with UMAP. Cell types were assigned based on established kidney markers, including proximal tubule (PT), collecting duct principal cells (CD_PC), loop of Henle (LOH), distal convoluted tubule (DCT), collecting duct intercalated cells A (CD_ICA) and B (CD_ICB), injured proximal tubule (IPT), endothelial cells (ENDO), complement factor H–associated cells (CFH), podocytes (PODO), mesangial cells (MES), and leukocytes (LEUK). Expression of prioritized genes was examined across cell types using DotPlot. Differences between groups were visualized using violin plots.

### Statistical analysis

All statistical analyses were performed using R (version 4.3.0). Differences between groups were assessed using the Wilcoxon rank-sum test. Correlation analyses between gene expression and clinical indicators were performed using Pearson or Spearman methods as appropriate. For scRNA-seq data, differential expression between groups within specific cell types was assessed using the Wilcoxon test implemented in the Seurat package. The R package ‘ggplot2’ was utilized for creating Manhattan plots, and ‘forestplot’ was used for generating forest plots. The code for generating SMRLocusPlot and SMREffectPlot was adapted from Zhu et al. [29].

## Results

### Lipid metabolism genes associated with DN

**Figure 1.**
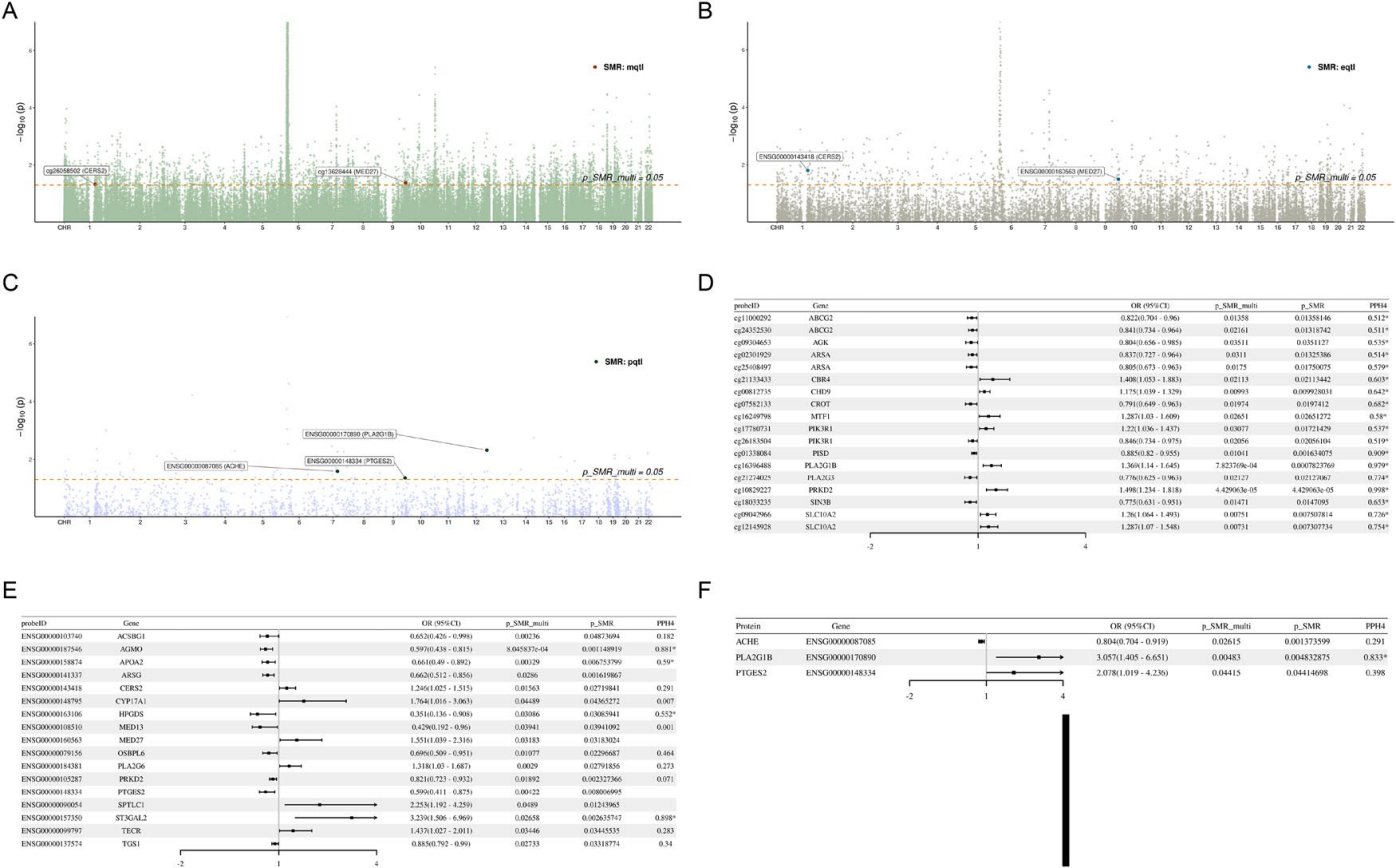
Multi-omics SMR analysis integrating blood mQTL, eQTL, and pQTL data identifies lipid metabolism–related genes associated with diabetic nephropathy (DN). (A–C) Manhattan plots showing the genome-wide SMR results integrating DN GWAS with blood-derived mQTL (A), eQTL (B), and pQTL (C) datasets. Each point represents an individual CpG site, gene, or protein tested for association with DN. The horizontal dashed line denotes the significance threshold (p_SMR_multi = 0.05). Significant loci are annotated, including cg26058502 (*CERS2*) and cg13628444 (*MED27*) in the mQTL layer, *CERS2* and *MED27* in the eQTL layer, and ACHE, PLA2G1B, and PTGES2 in the pQTL layer. (D–F) Forest plots summarizing the odds ratios (ORs) and 95% confidence intervals across mQTLs (D), eQTLs (E), and pQTLs (F). *PPH4 > 0.5.

To investigate the genetic mechanisms linking lipid metabolism to DN, SMR was performed by integrating blood QTLs with DN GWAS data. As shown in Figure 1(A–C), multiple loci were associated with DN risk. In the discovery stage, the mQTL analysis detected 90 CpG sites corresponding to 52 genes, with 18 loci from 14 genes demonstrating colocalization evidence. For example, the CpG site cg16396488 in *PLA2G1B* showed a positive association with DN risk, with colocalization evidence (OR = 1.369, 95% CI: 1.14–1.645, p_SMR = 7.82 × 10^−4^, PPH4 = 0.979; Figure 1(D)). The eQTL analysis identified 17 DN-associated genes, among which *AGMO, APOA2, HPGDS, and ST3GAL2* showed colocalization evidence (Figure 1(E)). The pQTL analysis further revealed three protein-level associations, including ACHE, PLA2G1B, and PTGES2. Among them, PLA2G1B, again, demonstrated colocalization evidence (OR = 3.057, 95% CI: 1.405–6.651, p_SMR = 0.005, PPH4 = 0.833; Figure 1(F)).

In the validation stage, five CpG sites from *ABCG2, GPX4,* and *MED27* were replicated in the GCST005881 dataset. For eQTL results, only *MED27* expression replicated, whereas no protein-level associations showed replication.

The complete SMR results from both discovery and validation stages are summarized in Supplementary Table S2–7. After removing overlapping genes between the eQTL and pQTL results (e.g., *PTGES2*), 19 genes underwent GO and KEGG pathway enrichment analyses.

### Lipid metabolism pathways and a core CERS2-MED27-PLA2G1B module associated with DN

**Figure 2.**
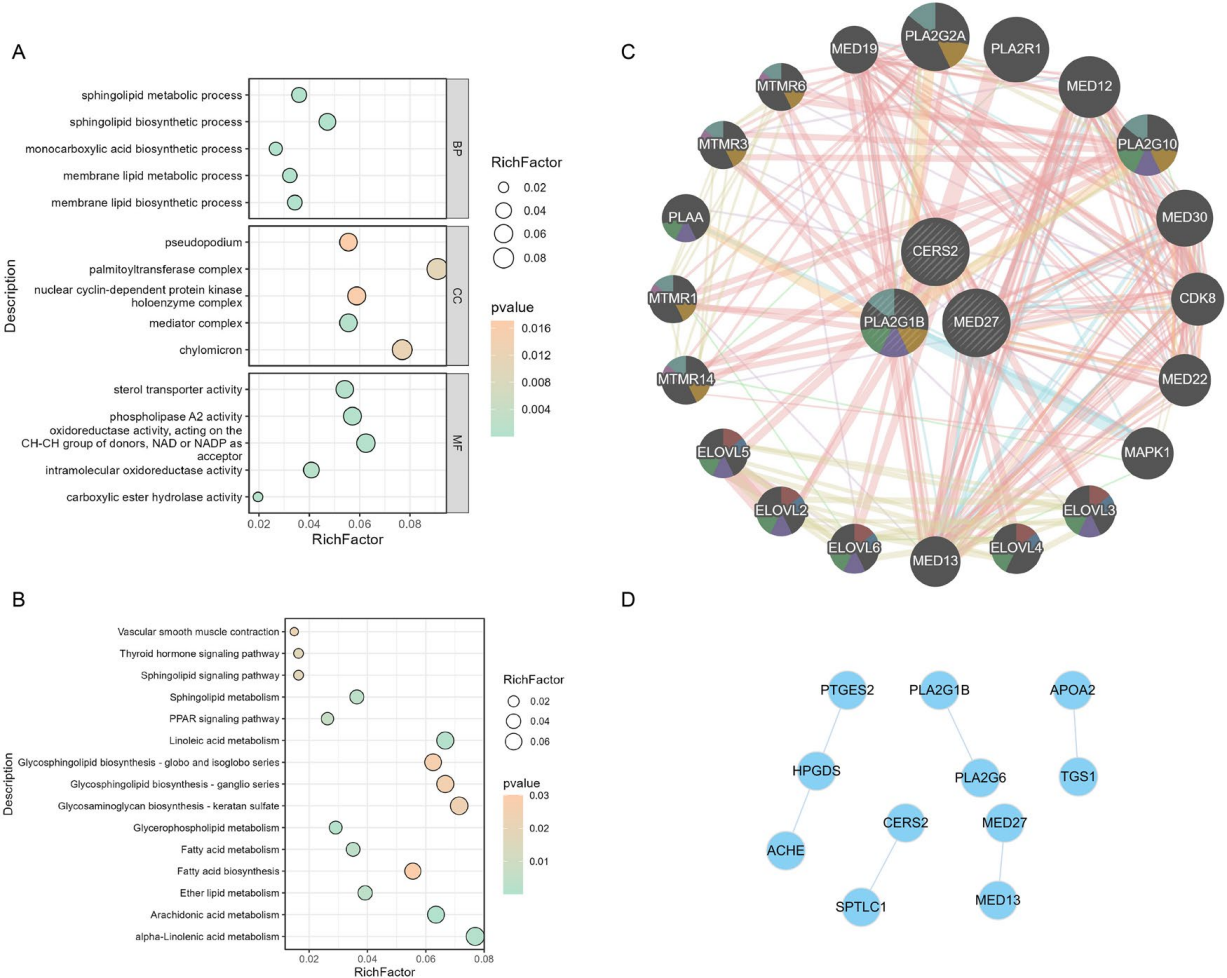
Functional enrichment and interaction network analyses of SMR-identified lipid metabolism–related genes associated with DN. (A) GO enrichment analysis of 19 genes prioritized by SMR, categorized by biological process (BP), cellular component (CC), and molecular function (MF). (B) KEGG pathway enrichment analysis showing significant involvement of these genes in lipid-related and signaling pathways. Bubble size represents the RichFactor, and color intensity indicates the adjusted p-value. (C) GeneMANIA illustrates a dense functional network centered on CERS2, MED27, and PLA2G1B. (D) STRING displays a core interaction module composed of ten proteins. Nodes represent genes; connecting lines denote molecular or functional interactions.

Functional enrichment further clarified the biological relevance of the 19 SMR-prioritized genes **(**Supplementary Table S8). The GO (Figure 2(A)) and KEGG (Figure 2(B)) results showed that these genes were significantly enriched in lipid metabolism-related biological processes and pathways, including sphingolipid metabolism, glycerophospholipid metabolism, fatty acid metabolism, and the PPAR signaling pathway. These pathways are known to regulate membrane lipid remodeling, energy homeostasis, and inflammatory signaling, all of which are related to the pathogenesis of DN.

To determine whether these genes function as a coordinated biological module, PPI network analysis was performed. The GeneMANIA network revealed a tightly interconnected functional cluster centered on CERS2, MED27, and PLA2G1B (Figure 2(C)), and the STRING network identified a core interaction module composed of 10 lipid metabolism–related proteins (Figure 2(D)). Collectively, these findings indicate that the SMR-identified genes form a functionally coherent network involved in lipid metabolic regulation and renal pathology.

### CERS2, MED27, and PLA2G1B are key genes associated with DN

To elucidate the molecular mechanisms linking genetic regulation to DN, integrative mQTL-eQTL analysis explored epigenetic-transcriptional regulation (Supplementary Table S9). Based on the instrument validity filtering (SMR + HEIDI), CERS2, MED27, and PLA2G1B were identified as valid cross-omics associations with DN, while PLA2G1B additionally demonstrated colocalization evidence (Table 1).

**Table 1.** Summary of multi-omics SMR results for *CERS2, MED27*, and *PLA2G1B* associated with diabetic nephropathy (DN), with colocalization evidence for selected loci.

Gene/Protein	Omics Level	Probe ID/CpG site	OR (95% CI)	p_SMR	p_SMR_multi	p_HEIDI	Colocalization Posterior (PPH4)
CERS2	mQTL	cg05630111	0.828 (0.696–0.985)	0.033	0.033	0.671	0.306
		cg13073141	0.817 (0.677–0.985)	0.035	0.035	0.470	0.305
		cg18611122	0.863 (0.746–0.999)	0.048	0.048	0.666	0.305
		cg22012583	0.816 (0.677–0.985)	0.034	0.034	0.716	0.304
		cg26058502	0.896 (0.803–0.999)	0.047	0.047	0.672	0.266
	eQTL	ENSG00000143418	1.246 (1.025–1.515)	0.027	0.016	0.725	0.291
MED27	mQTL	cg13628444	0.830 (0.693–0.993)	0.042	0.042	0.942	NA
	eQTL	ENSG00000184661	1.551 (1.039–2.316)	0.032	0.032	0.748	NA
PLA2G1B	mQTL	cg16396488	1.369 (1.14–1.645)	0.001	0.001	0.896	0.979
		cg22335246	1.281 (1.005–1.633)	0.046	0.046	0.968	0.36
		cg20313400	1.06 (1.004–1.12)	0.035	0.037	0.966	0.364
	pQTL	ENSG00000170890	3.057 (1.405–6.651)	0.005	0.005	0.022	0.833

Integration of mQTL, eQTL, and pQTL datasets with DN GWAS data identified three key genes showing significant genetic associations. The table summarizes the corresponding CpG sites or probes, effect sizes [OR (95% CI)], SMR significance levels (p_SMR and p_SMR_multi), heterogeneity test results (p_HEIDI), and colocalization posterior probabilities (PPH4). NA: not available.

**Figure 3.**
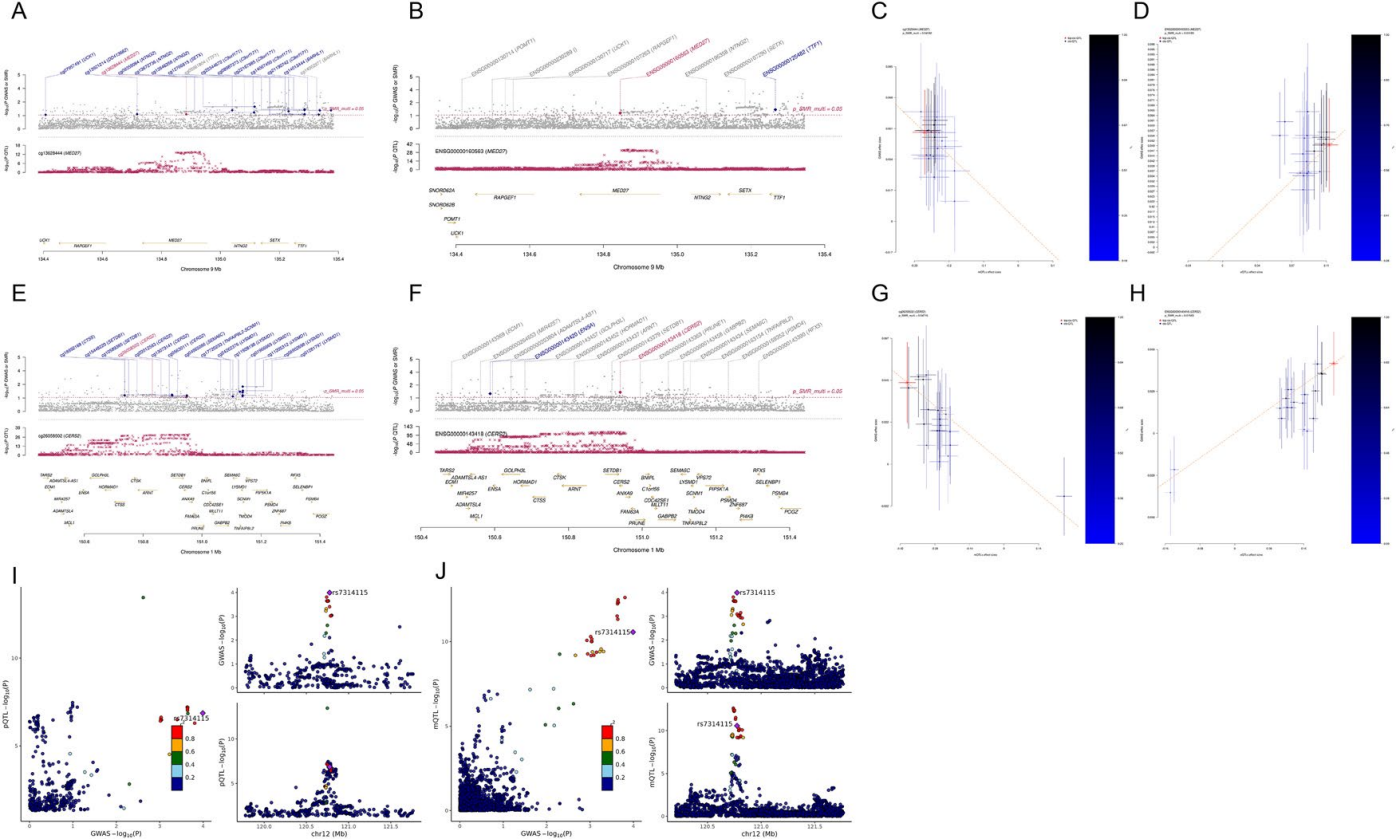
Integrative multi-omics SMR identifies *MED27, CERS2,* and *PLA2G1B* as key genes associated with DN, with colocalization evidence for *PLA2G1B*. (A–D) SMR locus (A, B) and effect (C, D) plots for *MED27*, integrating DN GWAS with mQTL (A, C) and eQTL (B, D) signals. (E–H) SMR locus (E, F) and effect (G, H) plots for *CERS2*, showing overlapping DN GWAS and QTL association signals at both methylation (E, G) and expression (F, H) levels. (I–J) Colocalization locus plots for *PLA2G1B*, showing the alignment of DN GWAS signals with those regulating *PLA2G1B* DNA methylation (mQTL; I) and protein abundance (pQTL; J). The lead variant rs7314115 shows overlap across datasets (r^2^ > 0.8, PPH4 > 0.8).

For *MED27*, the mQTL analysis identified a significant association between the methylation site cg13628444 and DN risk (OR = 0.83, 95% CI: 0.693–0.993, p_SMR = 0.04, p_SMR_multi = 0.04, p_HEIDI = 0.94; Figure 3(A,C)). Conversely, eQTL analysis demonstrated that increased *MED27* expression (OR = 1.551, 95% CI: 1.039–2.316, p_SMR = 0.03, p_SMR_multi = 0.032, p_HEIDI = 0.748) correlated with elevated DN risk (Table 1, Figure 3(B,D)). The integrative mQTL-eQTL SMR showed that cg13628444 methylation was negatively associated with *MED27* expression (OR = 0.662, 95% CI: 0.583–0.751, FDR = 3.82 × 10^−10^; Table 2). These findings suggest that hypermethylation of cg13628444 may suppress *MED27* expression, potentially mitigating DN risk.

**Table 2.** Significant epigenetic–transcriptional regulatory relationships among core genes revealed by mQTL–eQTL SMR analysis.

Methylation Site ID (Exposure)	Gene/Protein	OR (95% CI)	p_SMR	p_SMR_multi	p_HEIDI	FDR
cg26058502	CERS2	0.604 (0.548–0.665)	1.34 × 10^−24^	1.34 × 10^−24^	0.014736	1.61 × 10^−23^
cg13628444	MED27	0.662 (0.583–0.751)	1.59 × 10^−10^	1.59 × 10−¹⁰	0.07835384	3.82 × 10^−10^

SMR analysis integrating methylation and expression QTLs identified significant cis-regulatory effects of CpG methylation on gene expression for *CERS2* and *MED27*. The table lists the associated CpG sites, corresponding genes, effect sizes [OR (95% CI)], SMR significance levels (p_SMR, p_SMR-multi), heterogeneity (p_HEIDI), and FDR-adjusted p-values, demonstrating evidence for methylation-dependent transcriptional regulation. NA: not available.

For *CERS2*, five CpG sites (cg05630111, cg13073141, cg18611122, cg22012583, and cg26058502) exhibited significant associations with DN, all showing negative correlations with disease risk (OR range: 0.816–0.836). The representative locus and effect plots of cg26058502 are shown in Figure 3(E,G). eQTL analysis revealed that higher *CERS2* expression was associated with an increased DN risk (OR = 1.246, 95% CI: 1.025–1.515, p_SMR = 0.027, p_SMR_multi = 0.016, p_HEIDI = 0.725; Table 1, Figure 3(F,H)). Notably, cg26058502 displayed a significant negative association with *CERS2* expression in the mQTL-eQTL analysis (OR = 0.604, 95% CI: 0.548–0.665, FDR = 1.61 × 10–23; Table 2), suggesting a methylation-dependent regulatory mechanism.

In addition, *PLA2G1B* exhibited cross-omics colocalization evidence. The locus colocalization plots showed that the lead variant rs7314115 drove association peaks that aligned between the DN GWAS and both mQTL and pQTL datasets (Figure 3(I,J)). The consistent signal overlap and high LD (r^2^ > 0.8) indicate that the same genetic variant likely influences *PLA2G1B* methylation and protein abundance as well as DN susceptibility, providing statistical evidence for a shared variant.

Together, methylation-mediated transcriptional repression of *CERS2* and *MED27*, along with methylation- and protein-linked regulation of *PLA2G1B*, represent genetically supported associations that may contribute to DN pathogenesis.

### CERS2, MED27, and PLA2G1B expression correlates with improved renal function

**Figure 4.**
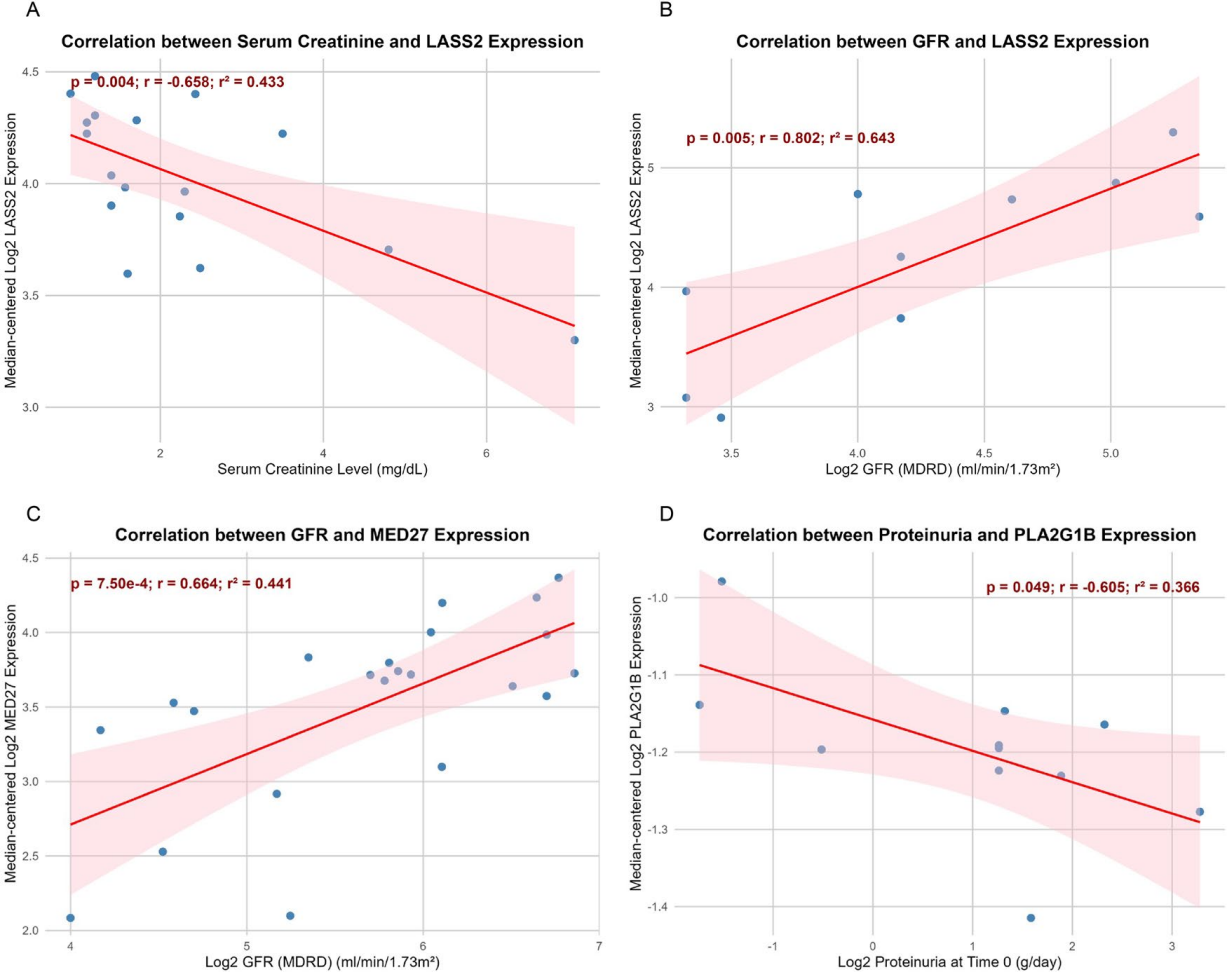
Nephroseq database validation of *CERS2, MED27,* and *PLA2G1B* expression in relation to renal function in DN. (A) Correlation of *CERS2/LASS2* expression with serum creatinine in the Ju CKD Tubulointerstitium (TubInt) dataset (*n* = 17). (B) Correlation of *CERS2/LASS2* expression with glomerular filtration rate (GFR) in the Woroniecka Diabetes Tubulointerstitium (TubInt) dataset (*n* = 10). (C) Correlation between *MED27* expression and GFR in the Woroniecka Diabetes Glomeruli dataset (*n* = 22). (D) Correlation between *PLA2G1B* expression and proteinuria in the Schmid Diabetes Tubulointerstitium (TubInt) dataset (*n* = 10).

To determine whether genetically associated genes exhibit consistent expression–phenotype relationships in renal tissue, Nephroseq datasets were analyzed in multiple kidney disease cohorts, including the Ju CKD Tubulointerstitium (TubInt) dataset (*LASS2* expression versus serum creatinine, *n* = 17), the Woroniecka Diabetes TubInt dataset (*LASS2* expression versus GFR, *n* = 10), the Woroniecka Diabetes Glomeruli dataset (*MED27* expression versus GFR, *n* = 22), and the Schmid Diabetes TubInt dataset (*PLA2G1B* expression versus proteinuria, *n* = 10). The expression of *CERS2* (recorded as *LASS2* in the database) showed a significant negative correlation with serum creatinine (*p* = 0.004, r = −0.658; Figure 4(A)) and a significant positive correlation with GFR (*p* = 0.005, *r* = 0.802; Figure 4(B)). Similarly, *MED27* expression positively correlated with GFR (*p* = 7.50 × 10^−4^, *r* = 0.664; Figure 4(C)). In addition, *PLA2G1B* expression demonstrated a significant negative correlation with proteinuria (*p* = 0.049, r = −0.605; Figure 4(D)). These data suggest that higher expression of *CERS2, MED27,* and *PLA2G1B* is associated with better renal function in DN tissue, contrary to the risk-increasing directions inferred from the SMR analyses. This discrepancy reflects differences between genetically inferred regulation and disease-state expression patterns.

### Stage- and cell-type-resolved expression patterns of CERS2, MED27, and PLA2G1B in DN

**Figure 5.**
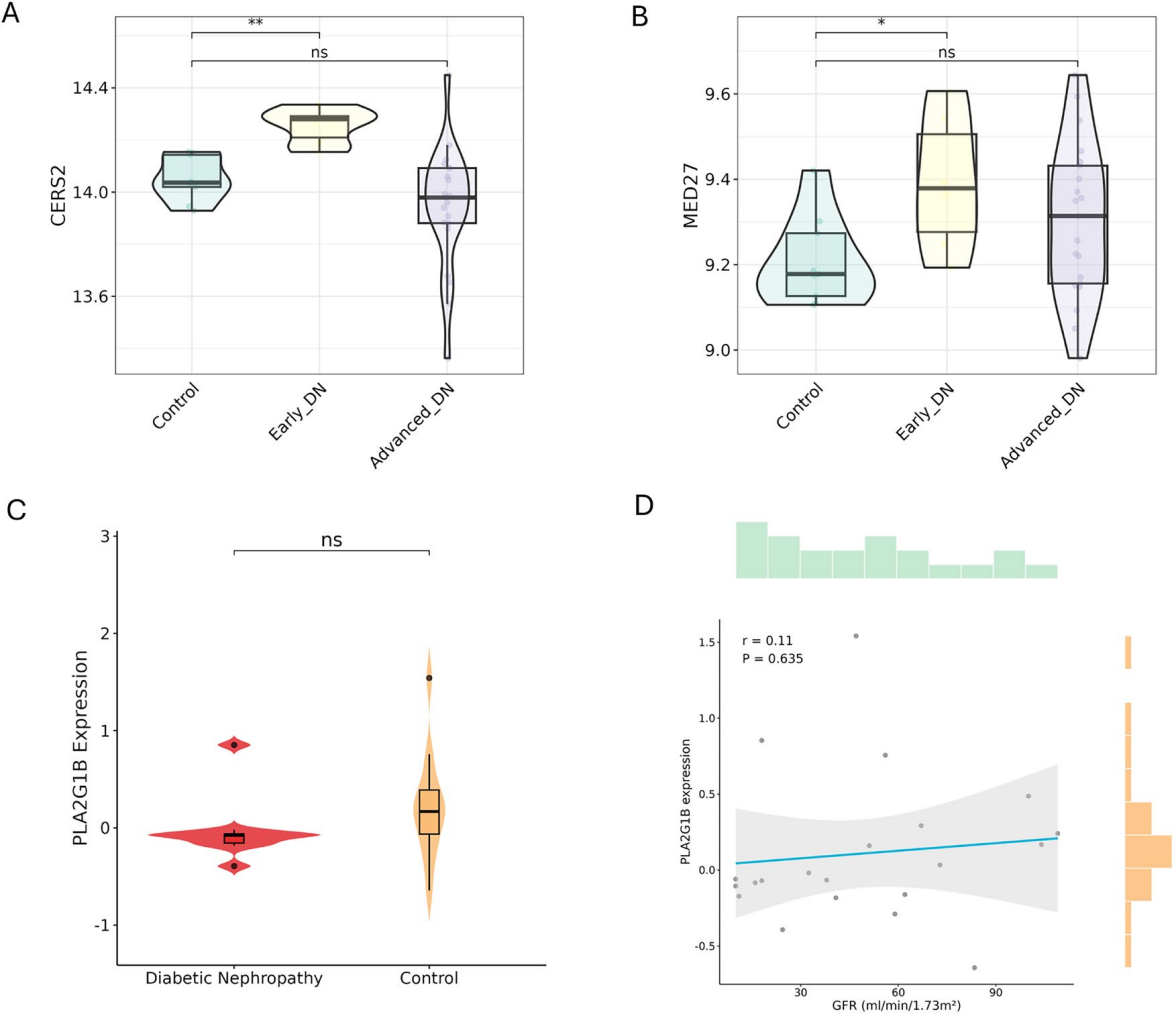
Stage-dependent expression patterns of *CERS2*, *MED27*, and *PLA2G1B* in DN. (A–B) Violin plots showing expression of *CERS2* (A) and *MED27* (B) in control (*n* = 9), early DN (*n* = 6), and advanced DN (*n* = 22) samples from GSE142025. (C) Comparison of *PLA2G1B* expression between DN and control samples in GSE30529. (D) Correlation between *PLA2G1B* expression and glomerular filtration rate (GFR) in GSE30529. *p < 0.05, **p < 0.01; ns, not significant.

To determine whether SMR associations reflect stage-dependent changes and cell-type-specific expression, bulk and single-cell transcriptomic datasets were analyzed. In GSE142025, *CERS2* and *MED27* expression significantly increased in early DN but not advanced DN (Figure 5(A,B)). In contrast, in GSE30529, *PLA2G1B* showed no difference between groups (Figure 5(C)) and no association with GFR (*r* = 0.11, *p* = 0.635; Figure 5(D)). These patterns are directionally consistent with Nephroseq but not with the SMR-inferred risk effects.

**Figure 6.**
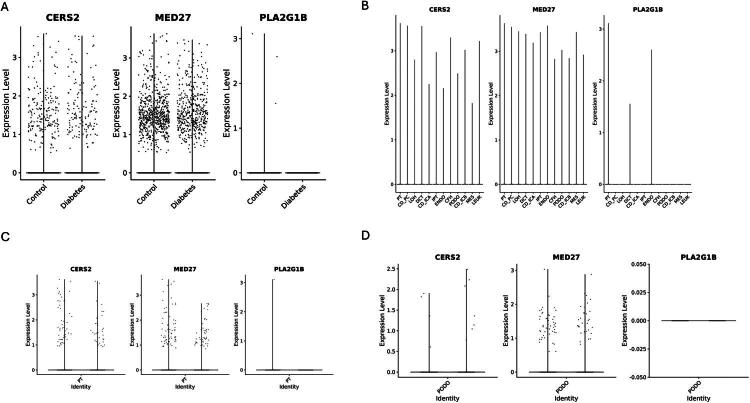
Cell-type-resolved expression of *CERS2*, *MED27*, and *PLA2G1B* in single-cell RNA-seq data from DN. (A) Violin plots showing expression of *CERS2*, *MED27*, and *PLA2G1B* between control and DN groups at the whole-sample level. (B) Bar plots illustrating average expression levels of *CERS2*, *MED27*, and *PLA2G1B* across annotated renal cell types, including proximal tubule (PT), collecting duct principal cells (CD_PC), loop of Henle (LOH), distal convoluted tubule (DCT), collecting duct intercalated cells A (CD_ICA) and B (CD_ICB), injured proximal tubule (IPT), endothelial cells (ENDO), complement factor H–associated cells (CFH), podocytes (PODO), mesangial cells (MES), and leukocytes (LEUK). (C–D) Violin plots displaying gene expression in PT (C) and PODO (D) subsets. Data were derived from GSE131882, including kidney cortex samples from controls (*n* = 3) and early DN (*n* = 3).

scRNA-seq further showed cell-type-specific expression patterns. Quality metrics were comparable across samples, t-SNE showed clear clustering without condition or sample separation, and UMAP demonstrated improved mixing after Harmony integration (Figure S2A–C). scRNA-seq resolved major renal cell populations (Figure S2D), supported by established marker genes (Figure S2E). Across cell types, *PLA2G1B* was barely detected, *CERS2* showed low expression without clear enrichment, and *MED27* was more evident in selected cell types, such as PODO and CD_ICA (Figure S2F). Across all samples, *CERS2* and *MED27* showed similar expression patterns between control and DN groups, while *PLA2G1B* remained minimally detected (Figure 6(A)). Across cell types, *CERS2* and *MED27* were detectable in multiple populations, whereas *PLA2G1B* signal was only observed in PT, DCT, and ENDO (Figure 6(B)). In PT and PODO subsets, *CERS2* and *MED27* signals were present without clear separation between groups, while *PLA2G1B* remained largely undetectable (Figure 6(C–D)). These findings show minimal disease-associated changes at the single-cell level and do not support the SMR-inferred risk direction, indicating discordance between genetic effects and observed expression patterns.

## Discussion

Multi-omics SMR analyses identified *CERS2, MED27,* and *PLA2G1B* as DN risk genes. However, tissue data from Nephroseq showed expression patterns linked to improved kidney function. Bulk and single-cell analyses further showed stage-dependent changes for *CERS2* and *MED27* and minimal disease-associated differences at the cellular level. This divergence emphasizes the complex regulatory roles of these genes in DN and warrants further investigation into their biological functions in disease development.

The opposite directions observed between SMR estimates and renal tissue expression highlight a distinction between genetic susceptibility and disease-stage transcription. SMR captures the effect of genetically regulated expression mediated by germline variants, whereas renal tissue expression reflects downstream changes in established DN. In the present study, bulk transcriptomic data showed that *CERS2* and *MED27* expression increased in early DN but not in advanced stages, while *PLA2G1B* showed no consistent changes or association with renal function. Single-cell analysis further showed low or restricted expression with no clear disease-associated shifts. These findings suggest that genetically predicted expression influences disease risk through early or systemic mechanisms that are not preserved in late-stage renal tissue. Therefore, blood-derived QTLs may capture upstream genetic effects on DN susceptibility, even when corresponding transcriptional changes are not observed in renal tissue. From a clinical perspective, these genes may inform genetic risk stratification, whereas their tissue expression may serve as markers of disease state or progression.

*CERS2* encodes ceramide synthase 2, an enzyme involved in the synthesis of very long-chain ceramide (VLC), which plays a vital role in lipid metabolism and cellular homeostasis. Genetic studies have associated *CERS2* variants with increased susceptibility to diabetes-related complications, including DKD [30,31]. Clinical data have also indicated that *CERS2* polymorphisms are associated with increased risk of DKD progression and all-cause mortality, although their predictive value remains modest compared with traditional clinical factors [32]. Functional studies have shown that alterations in VLC ceramide synthesis, induced by the E115A mutation or *CERS2* deficiency, impair energy expenditure, insulin sensitivity, and mitochondrial function [33,34]. Additionally, hepatic *CERS2* promotes the production of beneficial VLC ceramides, alleviating insulin resistance and steatosis [35]. The present study suggests that genetically predicted lower *CERS2* expression, inferred from blood methylation signals at cg26058502, may potentially reduce DN risk, highlighting regulatory effects that might differ from tissue-specific metabolic roles described in experimental systems. Future studies that incorporate kidney-specific QTL data and functional perturbation models will help clarify the kidney-related effects of *CERS2* regulation and determine whether *CERS2*-associated pathways can be used for risk stratification or targeted treatment in DN.

*MED27* encodes a subunit of the mediator complex, which facilitates RNA polymerase II recruitment, promoting transcription initiation and enhancer-promoter communication [36]. Although direct evidence linking *MED27* to DN is limited, emerging genomic studies have associated *MED27* variants with neurodevelopmental and metabolic disorders characterized by disrupted lipid homeostasis and energy regulation [37,38]. These observations suggest that *MED27* may affect transcriptional programs involved in metabolic and inflammatory pathways relevant to DN. In this study, SMR analysis showed that higher methylation at cg13628444, corresponding to lower *MED27* expression, was associated with reduced DN risk, implying that dampened *MED27*-mediated transcriptional activity may mitigate disease-promoting pathways by restricting transcriptional programs related to metabolic stress and inflammation. Given the limited prior evidence linking *MED27* to renal disease, these findings highlight a previously unrecognized transcriptional component that warrants further investigation in DN.

Regarding protein-level associations, the pQTL analysis identified PLA2G1B as positively associated with DN risk, supported by colocalization evidence in the discovery phase. PLA2G1B contributes to the production of lysophospholipids such as lysophosphatidylcholine. Experimental studies in mouse metabolic models have shown that loss of PLA2G1B activity improves lipid handling, enhances glucose tolerance, and protects against diet-induced metabolic dysfunction [39]. PLA2G1B has also been identified as a toxicity-related target in murine renal injury models, in which its activation leads to glycerolipid disturbances and kidney damage [40]. These data are consistent with the SMR finding that higher PLA2G1B expression may contribute to DN susceptibility. However, this association did not replicate in the validation cohort, necessitating further investigation in larger, independent datasets.

The comprehensive multi-omics SMR strategy offers advantages over traditional observational studies by utilizing genetic instruments and integrating epigenetic and proteomic layers. Nevertheless, several limitations should be acknowledged. First, while the primary analyses used large cohorts, the lack of consistent validation for some findings across all cohorts underscores the need for cautious interpretation and further replication. Second, the analyses primarily utilized blood-derived QTLs, as currently available kidney-specific QTL datasets have limited sample sizes and coverage, which constrain reliable SMR inference. As a result, these findings reflect genetically supported effects at the systemic level and may not fully reflect gene regulation in kidney tissue. In addition, the Nephroseq analysis is observational and based on bulk tissue data, and may be influenced by reverse causation, cell-type composition, and stage-dependent transcriptional remodeling. Although stage-stratified and single-cell analyses were performed, these factors cannot be fully excluded. Future studies incorporating kidney-specific QTL data would be beneficial. Third, while SMR and HEIDI tests help mitigate pleiotropy, and multi-omics integration was used to support prioritization, the limited colocalization evidence for some loci (e.g. CERS2 and MED27) implies that residual confounding by unmeasured factors or complex pleiotropic effects. cannot be entirely excluded. Fourth, the GWAS datasets originated predominantly from European populations, and the generalizability of the findings to other ancestries requires further evaluation. Last, downstream experimental validation using cellular and animal models is crucial to elucidate the precise biological mechanisms and confirm these genes as bona fide therapeutic targets. In future work, integrating multi-ancestry GWAS datasets, incorporating kidney-specific QTL layers, and performing mechanistic perturbation experiments targeting *CERS2, MED27, and PLA2G1B* in renal cell lines and DN animal models will help clarify their functional roles and therapeutic potential.

## Conclusions

*CERS2, MED27,* and *PLA2G1B* are lipid metabolism-related genes associated with DN risk. Bulk and single-cell analyses showed stage-dependent changes for *CERS2* and *MED27* and limited disease-associated expression changes at the cellular level. The contrasting directions between renal tissue expression and SMR estimates suggest differences between genetic susceptibility and disease-stage transcription. These genes represent genetically supported candidates for further functional investigation.

## Supplementary Material

new_Figure S2.tif

new_Figure S1.tif

## Data Availability

The supplementary tables supporting the findings of this study are openly available in the Zenodo repository. URL: https://zenodo.org/records/19688269 DOI: 10.5281/zenodo.19688269
