# Supplementary figures and images for: Integrative multi-omics Mendelian randomization reveals key lipid metabolism genes as therapeutic targets for diabetic nephropathy pathogenesis
Source: Ren Fail. 2026 May 31;48(1):2671460. doi: 10.1080/0886022X.2026.2671460 (PMC13228183; doi:10.1080/0886022X.2026.2671460)

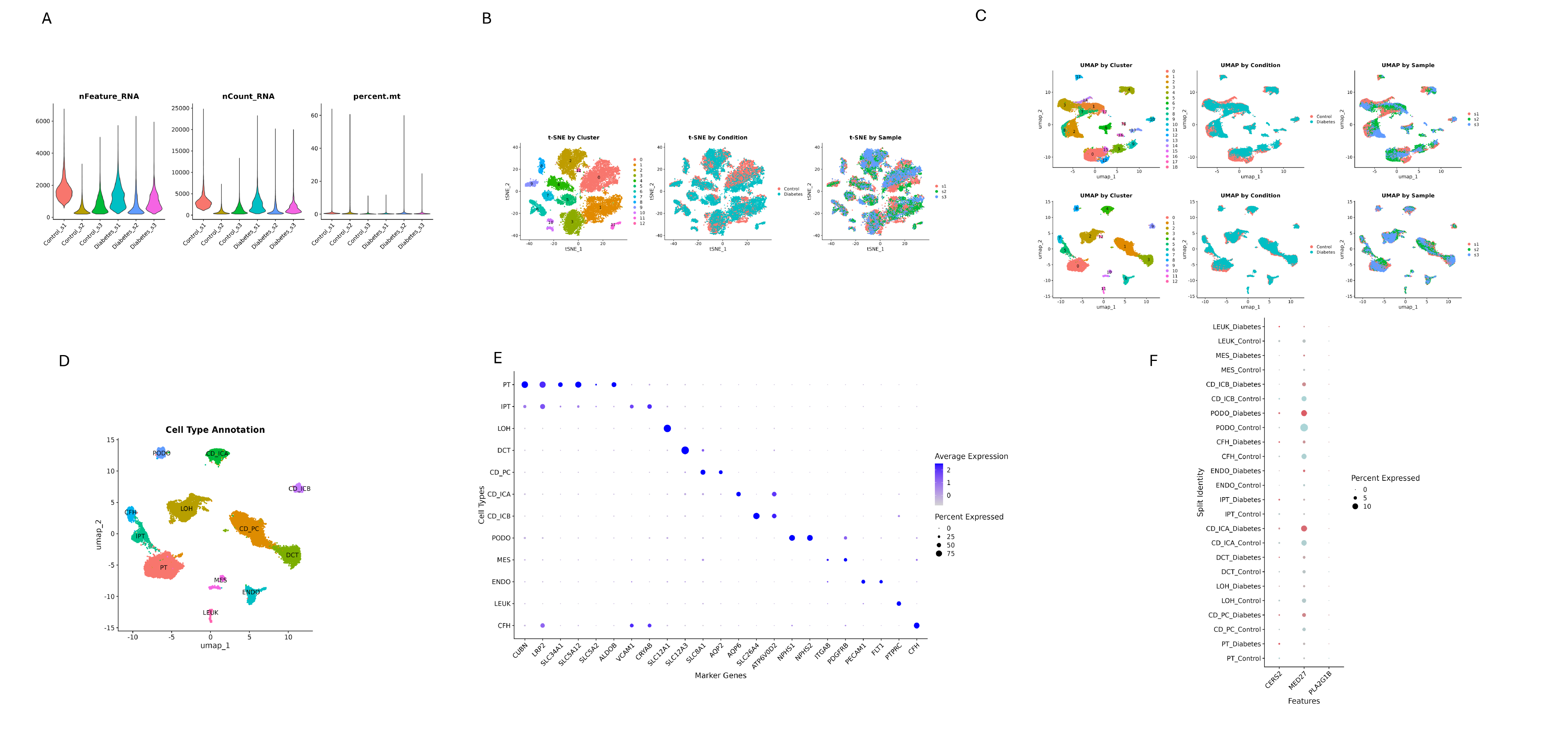

Supplement: new_Figure S2.tif [file IRNF_A_2671460_SM7611.tif]

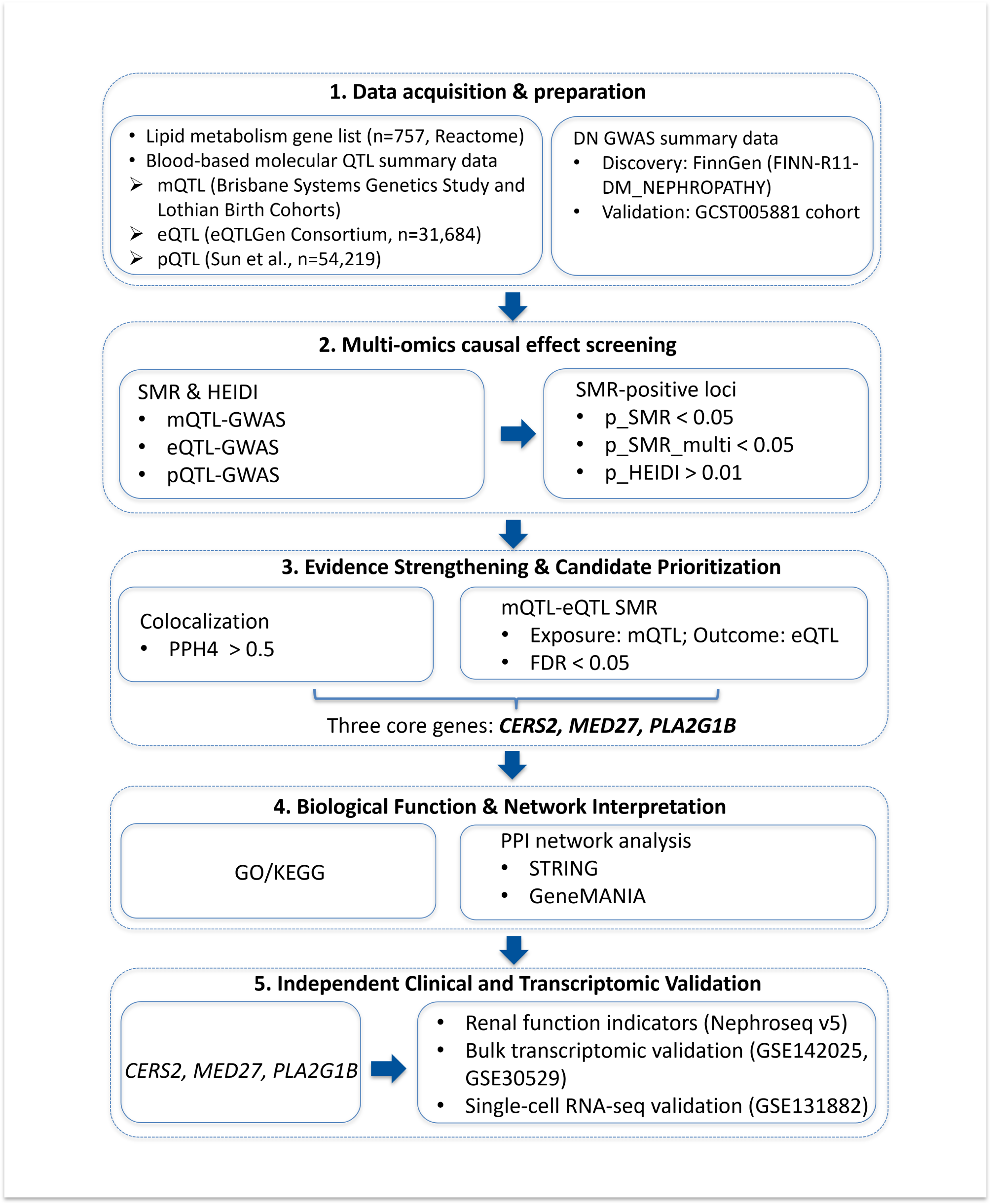

Supplement: new_Figure S1.tif [file IRNF_A_2671460_SM7610.tif]
